# Determination of no-observed effect level (NOEL)-biomarker equivalents to interpret biomonitoring data for organophosphorus pesticides in children

**DOI:** 10.1186/1476-069X-8-5

**Published:** 2009-02-19

**Authors:** Mathieu Valcke, Michèle Bouchard

**Affiliations:** 1 Direction des Risques Biologiques, Environnementaux et Occupationnels, Institut national de santé publique du Québec, Montréal, QC, Canada; 2 Department of Environmental and Occupational Health, Chair of Toxicological Risk Analysis and Management and GRIS, Université de Montréal, P.O. Box 6128, Main Station, Montreal, Quebec, H3C 3J7, Canada

## Abstract

**Background:**

Environmental exposure to organophosphorus pesticides has been characterized in various populations, but interpretation of these data from a health risk perspective remains an issue. The current paper proposes biological reference values to help interpret biomonitoring data related to an exposure to organophosphorus pesticides in children for which measurements of alkylphosphate metabolites are available.

**Methods:**

Published models describing the kinetics of malathion and chlorpyrifos in humans were used to determine no-observed effect level – biomarker equivalents for methylphosphates and ethylphosphates, respectively. These were expressed in the form of cumulative urinary amounts of alkylphosphates over specified time periods corresponding to an absorbed no-observed effect level dose (derived from a published human exposure dose) and assuming various plausible exposure scenarios. Cumulative amounts of methylphosphate and ethylphosphate metabolites measured in the urine of a group of Quebec children were then compared to the proposed biological reference values.

**Results:**

From a published no-observed effect level dose for malathion and chlorpyrifos, the model predicts corresponding oral biological reference values for methylphosphate and ethylphosphate derivatives of 106 and 52 nmol/kg of body weight, respectively, in 12-h nighttime urine collections, and dermal biological reference values of 40 and 32 nmol/kg of body weight. Out of the 442 available urine samples, only one presented a methylphosphate excretion exceeding the biological reference value established on the basis of a dermal exposure scenario and none of the methylphosphate and ethylphosphate excretion values were above the obtained oral biological reference values, which reflect the main exposure route in children.

**Conclusion:**

This study is a first step towards the development of biological guidelines for organophophorus pesticides using a toxicokinetic modeling approach, which can be used to provide a health-based interpretation of biomonitoring data in the general population.

## Background

Many studies have been published on the characterization of occupational exposure to several pesticides [1-7]. In the specific context of occupational exposure assessment to organophosphorus (OP) insecticides, toxicokinetic models have also been developed, which allow reconstruction of the amounts of an OP absorbed following an exposure episode in workers starting from cumulative amounts of urinary metabolites during specified time periods [8-11]. These models were used to predict cumulative urinary amounts of OP metabolites resulting from an exposure to a no-observed effect level (NOEL) dose, which were proposed as biological reference values (BRVs) for prevention in occupational health. Below these reference values, workers should not experience adverse health effects, whatever their exposure conditions, since OP toxicity is essentially of systemic nature. This approach was applied to different OP insecticides, namely azinphos-methyl [8], malathion [9], chlorpyrifos [10], and parathion [11].

In Bouchard et al. [7,9], model simulations under a variety of exposure scenarios showed that the lowest, thus most conservative, excretion values were obtained from a dermal exposure scenario with the slowest possible absorption rate rather than from respiratory or oral exposure scenarios. From these considerations, occupational BRVs were derived by simulating a dermal OP exposure such that the total absorbed daily dose corresponds to the absorbed NOEL. These guidance values were proposed in the form of total amounts of OP metabolites collected in urine over conveniently chosen time periods.

With regard to the assessment of environmental exposure to OPs in children, in agricultural or non-agricultural communities, several biomonitoring studies have been conducted [12-29]. However, dose estimates or health risks related to these environmental exposures, which mainly occur through the diet [30], were rarely assessed. Among the rare published studies on this aspect, one can cite that of Fenske et al. [26] in which dose estimates, reconstructed from biomarker concentrations using a simple calculation, were compared to the acceptable daily intake (ADI) of the World Health Organization (WHO). Using a simple calculation, Grandjean et al. [31] also determined the molar concentrations of alkylphosphates in urine (adjusted for the body weight) corresponding to an oral reference dose (RfD) for the most toxic pesticides, as a worst case scenario. The objective of the current study was to use a toxicokinetic modelling approach to determine NOEL – biomarker equivalents (NBE) to help provide a health-based interpretation of biomonitoring data from a previous study [22] on OP pesticide exposure assessment in children of a suburban area of the Province of Quebec, Canada.

## Methods

The proposed approach relies on the use of toxicokinetic models to simulate the amounts of alkylphosphate metabolites (APs), methyl and ethyl phosphates (MPs and EPs, respectively), excreted in urine over given time-periods that result from an absorbed NOEL dose under various hypothetical OP environmental exposures that may occur in children. These urinary amounts, defined as NOEL-biomarker equivalents (NBEs), were taken as reference values to which urinary biomarker measurements can be compared.

More specifically, in the present work, the MP and EP biological monitoring data reported by Valcke et al. [22] for each child and each void were expressed in cumulative amounts in nighttime urine collections per unit of body weight (BW) and compared to the different NBEs proposed for these OP metabolites. Briefly, a total of 442 complete first-morning urine voids were collected in children aged 3–7 during spring and summer, and MP and EP metabolites were measured in each sample. This sample was considered to represent approximately a 12-h urine collection assuming that the children urinated at bedtime, circa 7 pm, and woke up around 7 am.

For the determination of NBEs for MP and EP metabolites, the models published by Bouchard et al. [9,10] for malathion and chlorpyrifos (CPF) were respectively used. Assuming that the children urinated before bedtime around 7 pm and provided their urine sample around 7 am the following day, the models were run in order to simulate a 12-h nighttime cumulative urinary excretion, from 7 pm to 7 am, following three different daily OP exposure scenarios to NOEL absorbed doses. As was done in Bouchard et al. [9,10], these NOEL absorbed doses were derived from published human oral NOEL exposure doses for the inhibition of red-blood-cell acetylcholinesterase (RBC-AChE) activity of 16 mg/day (equivalent to 0.2 mg/kg BW-day) for malathion [32] and 0.1 mg/kg BW-day for CPF (Coulston et al., 1972, Institute of experimental pathology and toxicology, Albany Medical college). Simulations were thus conducted such that the total absorbed daily dose corresponded to the absorbed NOEL dose. The absorbed NOEL doses for malathion and chlorpyrifos were obtained by multiplying the published oral NOEL exposure doses by the absorption fraction determined in Bouchard et al. [9,10]. Corresponding cumulative amounts of urinary MP and EP biomarkers excreted during the 12-h nighttime period were then taken as NBEs.

Two oral exposure scenarios were used, since exposure was mainly attributed to OP residues in food [16,22,30], along with a dermal exposure scenario, which although maybe less realistic, has previously been shown to generate more conservative (lower) reference values [9,10,33]. The three exposure scenarios considered are as follows: 1) a bolus oral exposure at dinner (6 p.m.) such that the total daily absorbed dose corresponds to the absorbed NOEL dose and considering the oral absorption rate determined in Bouchard et al. [9,10]; 2) a bolus ingestion of one third of the NOEL at each of the 3 meals per day (7 a.m., noon, and 6 p.m.); 3) an 8-h dermal exposure starting at 7 am, that is at time t = 12 h preceding the onset of the nighttime urine collection period, such that the total absorbed daily dose corresponds to the absorbed NOEL dose and considering the dermal absorption rate reported in Bouchard et al. [9,10].

It is to be noted that the inhibition of RBC-AChE activity has been used in this report to assess NOEL-biomarker equivalents given that it has been reported as the reference biomarker of early cholinergic effect resulting from OP exposure [34-36]. The use of the NOEL for malathion reported by Moeller and Rider [32] is currently supported by Health Canada and that of chlorpyrifos reported by Coulston et al. (1972) is justified by the review of Zhao et al. (2006) on the reference dose for chlorpyrifos.

The proposed approach for the assessment of health risks was also compared to that of Fenske et al. [26], which consists of estimating, with a simple non-modeling approach, the absorbed daily doses of malathion and CPF from MP and EP biological measurements, respectively, and comparing these reconstructed doses to the WHO's ADI for these pesticides. The equations used from Fenske et al. [26] for the estimation of reconstructed doses are presented below:

(1) dd = []_creat. _× MW × DCER/BW

or

(2) dd = [] × MW × DUER/BW

where:

dd = Daily dose of the parent OP (malathion or CPF, in μg/kg-day)

[]_creat. _= Creatinine-adjusted urinary concentration of MP or EP, depending on the parent OP (μmol/g of creatinine)

[] = Urinary concentration of MP or EP, depending on the parent OP (in μmol/L)

MW = Molecular weight of the parent OP (malathion or CPF, in g/mol)

DCER = Daily creatinine excretion rate (g/day)

DUER = Daily urine excretion rate (L/day)

BW = Body weight (kg)

## Results

Table 1 presents the distribution of measured amounts of MP and EP metabolites in complete first morning voids. Table 2 presents the NBEs obtained with the model for MP and EP in 12-h nighttime collections, considering different daily exposure scenarios that may occur in children.

**Table 1:** Distribution of nighttime cumulative amounts of MP and EP metabolites of organophosphorus pesticides measured in the urine of children

Metabolite	Total amounts in nighttime collections (nmol/kg body weight)^a^							
	
	Geometric mean	Minimum	Maximum	Percentiles				
				
				5	25	50	75	95
Methylphosphates	2.05	0.006	75.4	0.201	0.849	2.10	5.12	15.8
Ethylphosphates	0.233	0.004	7.34	0.031	0.114	0.241	0.547	1.37
Methyl + ethylphosphates	2.43	0.011	77.0	0.306	1.09	2.39	5.66	17.3

Note: Methyl phosphates = the sum of dimethyl dithiophosphates (DMDTP), dimethyl thiophosphates (DMTP) and dimethyl phosphates (DMP); ethyl phosphates = the sum of diethyl dithiophosphates (DEDTP), diethyl thiophosphates (DETP) and diethyl phosphates (DEP).

^a ^Incomplete urine voids were excluded.

**Table 2:** NBEs for MP and EP in urine derived from model simulations of an exposure to a NOEL under different scenarios likely to occur in children

Exposure scenario used to derive the NBEs	Proposed NBE for methylphosphates in 12-h nighttime urine collections^a^ (nmol/kg bw)	Proposed NBE for ethylphosphates in 12-h nighttime urine collections^b^ (nmol/kg bw)
Ingestion of a bolus NOEL at dinner^c^	106	87
Ingestion of 1/3 NOEL at each meal^d^	127	52
8-h Dermal exposure to the NOEL^e^	40	32

^a ^Corresponds to the total amounts of methylphosphate metabolites in 12-h nighttime collections (from 7:00 pm to 7:00 am) following different simulated NOEL exposure scenarios.

^b ^Corresponds to the total amounts of ethylphosphate metabolites in 12-h nighttime collections (from 7:00 pm to 7:00 am) following different simulated NOEL exposure scenarios.

^c ^Simulation of an oral exposure to the NOEL of either malathion or chlorpyrifos at 6:00 pm.

^d ^Simulation of an oral exposure to 1/3 of the NOEL of either malathion or chlorpyrifos at 7:00 am, noon and 6:00 pm.

^e ^Simulation of an 8-h dermal exposure to the NOEL of either malathion or chlorpyrifos starting 12 h prior to the onset of urine sampling.

Given that ingestion of OP residues in foodstuffs generally appears as the main exposure route in children, oral NBEs for MP and EP metabolites were proposed on the basis of two different ingestion scenarios. NBEs of 106 and 127 nmol/kg BW for MP in 12-h nighttime collections were obtained when respectively considering i) an oral exposure to the NOEL of malathion at dinner or ii) an oral exposure to one third of the NOEL at each of the three daily meals (breakfast, lunch, dinner). With the same oral exposure scenarios, a CPF ingestion leads to corresponding NBEs for EP metabolites in 12-h nighttime collections of 87 and 52 nmol/kg BW. Considering that some OP exposures in children may also occur through the dermal route, dermal NBEs for MP and EP metabolites were also proposed for comparison purposes. When simulating an 8-h dermal exposure to the NOEL starting at time t = 12 h preceding the onset of urine collection, the malathion model from Bouchard et al. [9] yields a NBE of 40 nmol/kg BW for MP metabolites in 12-h nighttime urine collections. On the other hand, with the CPF model of Bouchard et al. [10], the same dermal exposure scenario leads to a NBE of 32 nmol/kg BW for EP metabolites in 12-h nighttime urine collections. Using a sensitivity analysis, it was however shown in Bouchard et al. [10] that a NBE based on a 12-h urine collection following the onset of a dermal exposure scenario is sensitive to the highly variable dermal absorption rate of CPF whereas a NBE based on a 12-h urine collection following an oral scenario is much less affected by variations in the oral absorption rate.

Table 3 presents the risk estimates obtained when comparing the measured amounts of MP and EP metabolites in urine with the proposed NBEs. These risk estimates correspond to the measured amounts of MP or EP metabolites in each urine sample divided by the proposed NBE for each of these metabolites. Given that some OP pesticides form MP metabolites while others are metabolized to EPs, risk estimates obtained on the basis of MP and EP measurements were also summed to evaluate the effects of combined exposures to MP and EP generating OPs. Risk estimates based on MP measurements were greater than those based on EP measurements, which is expected given that exposure to MP-generating OPs was on average nine times greater than exposure to EP-generating pesticides (Table 1). Table 3 shows that comparison of the observed amounts of MP and EP metabolites in each of the 442 provided urine samples with the proposed oral or dermal NBEs translates into risk estimates lower than 1 in all cases expect one, *i.e*. when comparing the measured MP value in one child with the dermal NBE for MP. In this one case, the MP-based risk estimate approaches the value of 2; consequently, this child was the only one to show a combined MP and EP risk estimate greater than 1.

**Table 3:** Risk estimates based on the comparison of observed MP and EP excretions with the different proposed NBEs

Scenario	Risk estimate^a^				No. of risk estimates >1
		
Metabolites considered	mean	SD	median	max	
**1- Comparison with the oral NBE derived from scenario 1^b^**					
Methylphosphates	0.04	0.06	0.02	0.71	0
Ethylphosphates	0.01	0.01	0.01	0.08	0
Sum of methyl+ethylphosphates	0.05	0.07	0.03	0.74	0
**2- Comparison with the oral NBE derived from scenario 2^c^**					
Methylphosphates	0.04	0.05	0.02	0.59	0
Ethylphosphates	0.01	0.01	0.01	0.14	0
Sum of methyl+ethylphosphates	0.04	0.06	0.02	0.63	0
**3- Comparison with the dermal NBE^d^**					
Methylphosphates	0.11	0.17	0.05	1.88	1
Ethylphosphates	0.02	0.03	0.01	0.23	0
Sum of methyl+ethylphosphates	0.12	0.17	0.06	1.92	1

^a ^The risk estimate corresponds to the ratio observed metabolite excretion/NBE.

^b ^Simulation of an oral exposure to the NOEL of either malathion or chlorpyrifos at 6:00 pm.

^c ^Simulation of an oral exposure to 1/3 of the NOEL of either malathion or chlorpyrifos at 7:00 am, noon and 6:00 pm.

^d ^Simulation of an 8-h dermal exposure to the NOEL of either malathion or chlorpyrifos starting 12 h prior to the onset of urine sampling.

Overall, mean risk estimates based on a comparison with the dermal NBE for MP and EP metabolites or the sum of MP and EP metabolites were lower than 0.2. When the comparison was made with an oral NBE, the highest mean risk estimate was 0.05, the maximum risk value was 0.74 and standard deviations were small indicating that the different individual risk estimate values were generally low.

Table 4 shows that risk estimates in the children under study were different when the approach followed by Fenske et al. [26] was used, in particular with regard to estimations based on MP measurements. With the approach of Fenske et al. [26], 130 out of the 442 individual risk estimates based on absorbed doses reconstructed from creatinine-adjusted MP concentrations were greater than 1, with a highest risk estimate of 29. When reconstructing the absorbed doses from volume-weighted MP concentrations, the numbers of individual risk estimates greater than 1 dropped to 22, with a highest risk estimate of 7.5. The individual risk estimates based on EP excretions were much lower. No individual risk estimate calculated from creatinine-adjusted EP concentrations was greater than 1 (max = 0.8); two of those calculated from volume-weighted concentrations were greater than 1 (max = 1.6).

**Table 4:** Risk estimates based on comparisons of reconstructed absorbed dose of malathion and chlorpyrifos with WHO's ADI

Scenario	Risk estimate^a^				No. of risk estimates >1
		
Metabolites considered	mean	SD	median	max	
**1- Comparison of the dose, reconstructed from creatinine-adjusted concentrations, with the ADI**					
Methylphosphates	1.10	2.18	0.51	29.1	130
Ethylphosphates	0.05	0.08	0.03	0.8	0
Sum of methyl + ethylphosphates	1.18	2.21	0.55	29.4	139
**2- Comparison of the dose, reconstructed from volume-weighted concentrations, with the ADI**					
Methylphosphates	0.30	0.55	0.13	7.5	22
Ethylphosphates	0.07	0.13	0.04	1.6	2
Sum of methyl + ethylphosphates	0.36	0.61	0.19	7.8	33

^a ^The risk estimate corresponds to the ratio of calculated dose, reconstructed from methyl and ethyl phosphate urinary excretion, divided by WHO's ADI.

## Discussion

This work used toxicokinetic models elaborated by Bouchard et al. [9,10] to describe the time-courses of malathion and CPF in humans in order to determine NOEL-biomarker equivalents (NBEs) under different oral or dermal exposure scenarios. It mainly contributed to provide biological reference values to help interpret biomonitoring data in order to facilitate public health decision-making related to the management of OP exposures in the general environment rather than in occupational settings as was done in the past [9,10].

Oral NBEs for MP and EP excretions based on two different exposure scenarios were determined in the present work along with a dermal NBE for MPs and EPs. The oral NBEs for MP and EP excretions are less conservative than the dermal NBE. The lower dermal NBE values result from the lower dermal absorption rate of malathion and CPF compared to the oral absorption rate [8,10], which translates into lower amounts of MP or EP metabolites in 0–12 or 0–24 h collections following the onset of an 8-h dermal exposure to the NOEL as compared to a bolus oral exposure. Given these kinetic considerations, the lowest oral NBE should provide a safe biological reference value below which the risk of cholinergic effects should be negligible in the general population, when exposure is most likely due mainly to the ingestion of OP residues on food. When the exposure scenario is not known or a dermal exposure is suspected, the use of the dermal NBE as a biological reference value corresponds to the more conservative approach, ensuring a certain margin of safety in the risk estimate.

The proposed biological reference values for MP and EP metabolites are based on the available models for malathion and chlorpyrifos. These models were developed with a given set of data and validated with a different set of data in orally and dermally exposed human volunteers. A good approximation of the experimental data was obtained in all cases ensuring the robustness of the models as detailed in Bouchard et al. [9,10]. Models for azinphos-methyl and parathion were also available, which allowed links to be made between an exposure or absorbed dose and MP or EP biomarker measurements, respectively. The reported human NOEL for azinphos-methyl (0.29 mg/kg/d) and parathion (0.058 mg/kg/d) is similar to that of malathion and chlorpyrifos (0.2 and 0.1 mg/kg/d, respectively) [8-11]. With the model for azinphos-methyl, the MP biomarker levels corresponding to the NOEL for this OP provided biological reference values slightly higher albeit in the same range (154, 139, 63 nmol/kg BW) as the ones obtained using the model for malathion (106, 127, 40 nmol/kg BW). The slightly higher biological reference values for azinphos-methyl despite a slightly lower NOEL are related to variations in the toxicokinetics of the two substances. As for the model for parathion, it was considered less appropriate than the model for CPF for modeling EP biomarker levels given that there appears to be a delay in the renal excretion of EP metabolites of parathion presumably due to para-nitrophenol metabolites of this OP [11,33].

Obviously, the children under study could theoretically have been exposed to other potentially more toxic OP pesticides than malathion and chlorpyrifos. However, according to the last survey on estimated average daily intakes of pesticide residues in Canadians [37], malathion, azinphos-methyl, chlorpyrifos, methylparathion, phosmet and phosalone were the OP pesticides to which the general population was mostly exposed through the diet. More recent unpublished data, collected at the time the children under study were sampled, tend to confirm this trend [38]. For methylparathion, phosmet and phosalone, no toxicokinetic model are yet available, but a human NOEL of 0.3 mg/kg/d has been reported for methylparathion, similar to that of malathion and azinphos-methyl, and a rat NOEL of 0.2 and 2 mg/kg/d was established for phosalone and phosmet, respectively [39]. As a result, an important contribution of significantly more toxic OP pesticides to the measured AP metabolites in the children under study appears unlikely.

Nonetheless, the children under study may have been exposed concurrently to several OPs by different routes (although oral exposure is the most likely route-of-entry). To account for possible multiple exposure routes, the more conservative NBE resulting from the dermal exposure scenario can thus be used, as mentioned earlier. Concomitant exposure to several OP pesticides was also accounted for in our evaluation since EP and MP metabolites are non-specific OP metabolites and thus respectively result from the biotransformation of all EP and MP-producing OP pesticides. Calculating risk estimates from the sum of EP and MP metabolites rather than MP alone did not result in an increased number of values greater than 1.

It was also found from model simulations that the derivation of NBEs considering a single daily exposure rather than a repeated daily exposure also contributes to provide safer NBE estimates. This is explained by the increase in daily urinary excretion of MP and EP metabolites, expressed as a fraction of the absorbed daily dose, during a repeated daily exposure, as can be shown from model simulations.

It is to be noted that the present study proposes biological reference values for non-specific metabolites of OP pesticides measured in urine. Estimating the risk resulting from an exposure to a given pesticide based on urinary measurements of specific metabolites is generally more accurate in the case of occupational exposure where the pesticide involved can be easily identified. Conversely, the proposal of biological reference values for non-specific OP metabolites rather than specific metabolites appears more accurate for environmental risk assessment of OP exposure where the pesticides to which subjects are exposed are unknown. Although some of the measured non-specific metabolites could stem from environmental degradation products already present in food [40], this would result in an actual overestimation of the human exposure to the parent compounds and thus of the human health risks. Indeed, toxicological activity of OP pesticides results from the oxon metabolite generated by the breakdown of the parent compounds, and the AP metabolites that are produced as by-products do not have toxicological activity.

With the derived NBEs, the toxicological risks attributed to OP exposures in a group of children were assessed using MP and EP measurements in several first-morning urine voids. On the basis of our approach, that is the comparison of the observed excretion values of MP and EP metabolites in children with the proposed NBEs, the children under study should incur a negligible risk of cholinergic effects related to OP pesticide exposure. Indeed, only one sample presented a risk estimate greater than 1 (1.88 on the basis of MP excretion and 1.92 on the basis of MP + EP excretion, when compared to a dermal NBE). In comparison, the results obtained by using the method of Fenske et al. [26] generated more concerning results (see Table 4). However, their method consists of comparing a calculated biologically-based dose estimates with the ADI used at the time [39]. In the case of malathion and CPF, the ADI is based on a human NOEL (0.2 and 0.1 mg/kg/d, respectively) to which a 10-fold uncertainty factor was applied to account for inter-individual variations within a population [39]. In our study, when considering MP-based risk estimates, a difference of approximately one order of magnitude was found between the risk estimates obtained with the NOEL-biomarker equivalent approach and those calculated from the comparison of an estimated absorbed dose of malathion and CPF and their ADI. This difference of one order of magnitude in risk values between the two approaches may stem from the use of a 10-fold uncertainty factor for interindividual variations when establishing the ADI for malathion. Indeed, the toxicokinetic models used to obtain the NBEs were elaborated and validated with human data and the human NOELs used in model simulations were the same as those considered for the derivation of the ADI; however, no uncertainty factor for interindividual variations was introduced with our kinetic approach.

The NBEs for cholinergic effects are still proposed with a margin of safety since the NOEL used to derive biomarker equivalents were based on the inhibition of red blood cell acetylcholinesterases (AChEs); these AChEs are more sensitive to OP inhibition than nervous system AChEs [41], responsible for the neurotoxic effects of OPs. The proposed NBEs were also based on a NOEL instead of a lowest-observed effect level (LOEL) [8,10]. Thus, an exposure exceeding a NOEL-based reference value does not imply that it exceeds the minimum dose required to produce an adverse effect. In our analysis of the studied children, since only one out of the 442 risk estimates exceeded the value of 1 and did not reach the value of 2, it appears unlikely that the exposure doses in the children under study exceeded the LOEL dose. Indeed, Dourson et al. [42] evaluated that the difference between a LOEL and a NOEL is at least two-fold 75% of the time.

Uncertainties in our NBE estimates are nevertheless related to the fact that the models used to develop our NBEs were elaborated using data in adults rather than in children. Although children are generally considered potentially more exposed to pesticides than adults, partly due to their specific behaviors (hand-to-mouth behavior, crawling) and physiological characteristics (higher dermal permeability) [43,44], for a given molar absorbed dose per unit of body weight, evidence that children of the age group under study (3–7 years old) are more sensitive than adults, on a toxicodynamic basis, remains to be confirmed. On the other hand, this does not preclude the possibility of kinetic differences between children and adults [45]. Interindividual variations in the inhibition capacity are also to be expected. If a default uncertainty factor of 10 was added to account for inter-individual variability or potential kinetic or sensitivity differences between adults and children, 138, 45 and 35 out of the 442 samples collected would respectively present MP values exceeding the reference value for the dermal exposure scenario and the two oral scenarios. In the case of EPs, corresponding values would be 5, 3 and 0. These values are similar to those obtained using the method of Fenske et al. [26]. However, Zhao et al. [36] reported that the necessity of applying this factor has to be put into perspective since the results of Mattsson et al. [46] unequivocally show that neonatal and young animals are equally or perhaps less sensitive than adults to ChE inhibition on a tissue dose and tissue response specific basis. Also, from the results of the study of Zheng et al. [47], it appears that neonatal experimental animals are not more sensitive than adults to repeated exposure to chlorpyrifos. In any case, Zhao et al. [36] stated in their review that an uncertainty factor of 10 was deemed sufficient to account for the overall uncertainty in the fairly large database used to assess a reference dose for chlorpyrifos.

In the models for malathion and chlorpyrifos, there is also an uncertainty in the fraction of the exposure dose considered to be recovered in the urine as MP or EP metabolites, when applied to a large group of children. A lower fraction than the one used in the model would have generated more conservative NBEs, hence with a direct impact on the number of studied children with AP excretions exceeding the biological reference values.

Other uncertainties are linked to the fact that acetylcholinesterase inhibition was considered the critical effect although effects appearing at lower doses, such as neurobehavioral effects [48,49], cannot be excluded. In particular, Grandjean et al. [31] reported an increased reaction time after a visual and auditory stimulus in children exposed to OP levels that were not associated with an inhibition acetylcholinesterase activities in red blood cells. The urinary levels measured in the children evaluated in the current study [22] were 5–6 times greater than those measured by Eskenazi et al. [49] in children for which developmental effects were reported to be positively associated with urinary AP metabolites, although with limited evidence.

It should also be noted that the risk of OP exposure was characterized in the current study from complete single urine voids, which are easy to obtain. A 24-h urine collection, although less practical, would have been a better sampling strategy given the large influence of the absorption rate on excretion during the first hours following an exposure [9,10]. Therefore, it cannot be excluded that the pattern of risk estimates might have been different, on the basis of 24-h urine collections.

## Conclusion

In conclusion, observed MP and EP excretions in children were compared with NOEL-biomarker equivalents determined using published toxicokinetic models. This toxicological approach indicated that the children under study generally exhibited biological OP levels below the derived NBEs. The proposed oral and dermal biological reference values for MP and EP excretions can be used to help interpret biomonitoring data in other populations where nighttime cumulative urinary excretions are collected.

## Abbreviations

AChE(s): Acetylcholinesterase(s); ADI: Acceptable Daily Intake; AP(s): alkylphosphate metabolite(s); BRV(s): Biological reference value(s); CPF: chlorpyrifos; RfD: Reference dose; EP(s): ethylphosphate metabolite(s); LOEL: Lowest-observed effect level; MP(s): methylphosphate metabolite(s); NBE(s): NOEL-biomarker equivalent(s); NOEL(s): No-observed effect level(s); OP(s): organophosphorus; WHO: World Health Organization.

## Competing interests

The authors declare that they have no competing interests.

## Authors' contributions

MV contributed to write the manuscript, contributed to the NBE approach and performed the comparison with measures of urinary metabolites in the children under study. MB contributed to write the manuscript, conceived the NBE approach and performed the modeling.
